# Napari-3D-Counter: A manual cell counter for napari

**DOI:** 10.21105/joss.09091

**Published:** 2026-03-15

**Authors:** Peter Newstein

**Affiliations:** 1 University of Oregon, Eugene, United States; 2 Howard Hughes Medical Institute, Eugene, United States

## Abstract

A common task across biological fields is to quantify the number of objects in an image. Often, the most efficient solution to this problem is to have an expert manually count those objects. This package, Napari-3D-Counter, includes the Count3D widget, a user-friendly interface to allow an expert to quickly count objects in 2D or 3D images visualized using napari, as well as auxiliary plugins that help to integrate Count3D into upstream and downstream analyses. Napari-3D-Counter focuses on being user-friendly for beginners and experienced users, and has been continually updated since its 2023 release. The package is available on both PyPI and conda-forge, and is indexed on napari-hub.

## Statement of need

Despite the many high-quality automated methods for identifying objects in images, expert annotation is sometimes the most practical option. For example, training and optimizing a machine learning model may require more effort than manual annotation. In these cases, user-friendly software is especially important to save time for the expert annotator. User-friendliness includes both ergonomics (whether the software is intuitive and efficient to use) and stability (whether the software works as expected).

I introduce the package Napari-3D-Counter, which leverages the Python / napari ecosystem to create a user-friendly interface for manual cell counting. Napari is a user-friendly multidimensional image viewer that is open source and implemented in the Python programming language (Sofroniew et al., 2025). Napari’s implementation language gives it the advantage of easily integrating with Python’s numerous scientific tools through a plugin system.

Because napari is under active development, upstream changes can affect plugins. To keep Napari-3D-Counter reliable, fixes are released promptly with the aid of unit tests, which cover over 90% of the code. These tests are automatically run before publication using a GitHub Action.

The functionality of the main widget provided by Napari-3D-Counter, Count3D, is similar to the FIJI cell counter plugin (De Vos, 2010), with important differences: no macros are necessary for keyboard automation, locations can be saved in the CSV format instead of XML (enabling easier integration with GUI spreadsheet tools) and most importantly, integration with the Python / napari ecosystem. This integration is significant because napari provides advanced 3D visualization capabilities, while the Python ecosystem offers powerful packages such as scikit-image (Walt et al., 2014) and SciPy (Virtanen et al., 2020) for analysis.

Native napari Points layers can replicate many of the core features of Count3D. However, Count3D has the advantage of being specifically specialized to count cells of different types: it takes one keyboard shortcut to switch between various cell type counters, and there is a live display of how many cells of each type have been counted. Furthermore, saving and loading a single CSV file containing the coordinates of all cells from all types is preferable to creating separate files for each type. Overall, using Napari-3D-Counter is likely to save the expert annotator’s time over using native Napari Points.

Finally, Count3D’s functionality is also similar to the manual spots feature of Imaris (Imaris, 2024). In addition to the ergonomic benefits of a bespoke cell counter listed above, a clear advantage of Count3D over Imaris is its availability under a free software license, while Imaris is proprietary software that requires a costly license.

Beyond Count3D’s core features, other functions related to manual cell counting are implemented in auxiliary plugins: IngressPoints, SplitOnShapes, and ReconstructSelected. IngressPoints takes a native napari points layer, perhaps created by automated labeling, and turns them into a counted cell type in Count3D. SplitOnShapes splits labels of cell types based on spatial information. For example, if a user wants to quantify the distribution of cells of multiple types across a tissue with multiple repeating segments (eg. spinal cord), they can use a napari Shapes layer to define all the segments in the X and Y axes, and SplitOnShapes will return a count of each cell type within each shape. Finally, ReconstructSelected can be used to aid in visualizing cells: if a user has a Label layer labeling all cells, but they only want to visualize a subset, ReconstructSelected will take those labels containing a Count3D cell and create an image layer containing only those cells, which can then be used to create 2D or 3D images. Overall, these auxiliary plugins help to integrate Count3D into more complex, semi-automated cell counting processes.

The utility of this plugin is also reflected in its use. It has been used in scientific publications (Drake et al., 2025; Sato et al., 2025) and has over 15,000 downloads on conda-forge.
